# Pre Conference Workshops, June 5

**DOI:** 10.3402/ejpt.v4i0.21269

**Published:** 2013-06-04

## PRE CONFERENCE WORKSHOPS, JUNE 5

## Full Day Workshops

### ESTD Workshop “From trauma through dissociation to psychosis: understanding and treating psychotic symptoms from a trauma and dissociation perspective”

#### A. Moskowitz Aarhus University, Denmark

Over the past decade, considerable research has linked traumatic experiences, including childhood trauma, to the development of psychotic symptoms. More recently, the concept of dissociation has been connected to psychosis, particularly auditory hallucinations, as well as to the historical conception of schizophrenia. In this workshop, Professor Andrew Moskowitz, the lead editor of “Psychosis, trauma and dissociation” will discuss historical, empirical, theoretical and clinical links between the concepts of trauma, dissociation and psychosis, with particular emphasis on: (1) connections between dissociation, Bleuler's schizophrenia and Kurt Schneider's first rank symptoms, (2) diagnostic issues between PTSD and psychotic disorders, (3) research evidence linking trauma with the development of delusions and dissociation with the development of auditory hallucinations and (4) clinical approaches to work with delusions and hallucinations informed by a trauma/dissociation perspective. Participants should come away with an increased understanding of the relevant concepts of trauma and dissociation to understand psychosis and an awareness of clinical approaches to work with psychotic symptoms from a trauma/dissociation perspective.

### Seeking Safety: a model for trauma and/or substance abuse

#### L. M. Najavits^1,2^

^1^Boston University School of Medicine, Boston, MA, USA; ^2^Harvard Medical School, Boston, MA, USA

The goal of this presentation is to describe “Seeking Safety”, an evidence-based treatment for trauma and/or substance abuse. We will cover (1) background on trauma and substance abuse (rates, presentation, models and stages of treatment, clinical challenges) and (2) implementation of “Seeking Safety” (overview, evidence-based). Assessment tools and community resources are also described. By the end of the training, participants can implement “Seeking Safety” in their setting. Learning methods include powerpoint, video, exercises, and discussion.

Objectives

(1) To review research and clinical issues in treating trauma and substance abuse.

(2) To increase empathy and understanding of trauma and substance abuse.

(3) To describe “Seeking Safety”, an evidence-based model for trauma and/or substance abuse.

(4) To provide assessment and treatment resources.

## Morning - Half Day Workshops

### Narrative exposure therapy as treatment for trauma spectrums disorders—basics

#### M. Schauer^1^ and M. Ruf-Leuschner^2^

^1^Center of Excellence for Psychotraumatology, University of Konstanz, Konstanz, Germany; ^2^University of Konstanz, Konstantz, Germany

The workshop gives an introduction to narrative exposure therapy (NET), a treatment for trauma spectrum disorders in survivors of multiple and complex trauma. NET builds on Brewin's theory of the dual representation of traumatic memories. It is thought to contextualize particular associative elements of the fear network, the sensory, affective and cognitive memories of trauma. It is therefore important to understand and process the memory of a traumatic event in the course of the particular life of a client. Therefore, in NET, the patient, with the assistance of the therapist, constructs a chronological narrative of his life story with a focus on the traumatic experiences. Fragmented reports of the traumatic experiences will be transformed into a coherent narrative. Empathic understanding, active listening, congruency and unconditional positive regard are key components of the therapist's behaviour. For traumatic stress experiences, the therapist asks in detail for emotions, cognitions, sensory information, physiological responses and probes for respective observations. The patient is encouraged to relive these emotions, while narrating without loosing the connection to the “here and now”: using permanent reminders that the feelings and physiological responses result from memories, the therapist links the experiences to episodic facts, that is time and place. By reprocessing in this way, meaning-making and integration is facilitated. At the end of treatment, the recorded autobiography may be used for human rights advocacy. The method of narrating the entire life story does not require the clients to select a single traumatic event from their trauma history. NET allows reflection on the person's entire life as a whole, fostering a sense of personal identity. Working through the biography highlights the recognition and meaning of interrelated emotional networks from experiences, facilitating integration and an understanding of schemas and behavioural patterns that evolved during development. Regaining of survivor's dignity and satisfaction of the need for acknowledgement as well as the explicit human rights orientation of “testifying” distinguishes the approach. The procedure is straightforward and can be easily understood by local therapists and counsellors in resource-poor contexts (i.e. after war and disaster). Additionally, the fact that the survivor receives a written biography as a result of the treatment has turned out to be a major incentive to complete the treatment. The procedure is demonstrated during the workshop with the involvement of participants.

### Trauma-related disorders in forensic settings: research, treatment and policy ESTSS FORENSIC TASK FORCE WORKSHOP

#### V. Ardino^1^, V. Caretti^2^, T. Elbert^3^ and A. Forrester^4^

^1^London School of Economics and Political Science, London, UK; ^2^University of Palermo, Palermo, Italy; ^3^Department of Psychology, University of Konstanz, Germany; ^4^South London and Maudsley NHS Foundation Trust and Institute of Psychiatry/King's College London, London, England, UK

Workshop presentation

This workshop introduces attendees to the main psychological and criminological issues underlying the definition, measurement and treatment of childhood violent trauma and trauma-related disorders in diverse forensic settings with relevant reference to research, treatment and service delivery. The presenters derive their expertise from work in forensic settings and from studies of perpetrators in war zones. Prospective and retrospective studies on children who were abused or neglected uncovered a high incidence of later delinquency. Moreover, children clinically referred to residential treatment with a history of abuse scored significantly higher on measures of reactive and verbal aggression than non-abused control children. Finally, a large proportion of homicide offenders come from unfavourable home environments and up to 80% of subjects within delinquent samples report witnessing of violence in their childhood or adolescence. We can differentiate two basically different forms of human aggressive behaviour: (1) The reactive-impulsive form appears in response to acute threat. Aggression may reduce the threat and with it negative valence and emotional arousal. (2) In contrast, appetitive aggression frequently starts with a low arousing state, but becomes both increasingly pleasurable and arousing with practice. It is planned, goal-directed, and, as we suspect, motivated by the pleasure of hunting and power. Appetitive aggression is the one of the powerful attackers and hunters. This workshop explains these concepts and practice forms of assessment. This workshop introduces attendees with specific instruments to assess childhood adversity and the attraction to violence in offenders. This workshop also presents a road map of criminal justice systems, including prisons, courts, probation services and police custody arrangements. This is important given the established evidence that demonstrates a considerable excess of all mental disorders within prison systems, particularly amongst pre-sentence (remand) populations, and evidence that the prison population is continuing to rise across all of the world's continents. With mounting evidence that some parts of criminal justice systems operate as mental healthcare triage areas, pathways to healthcare from criminal justice will be explored. Service access and availability issues will be introduced and the nature of assessment and treatment in environments that are often geared towards detention (and punishment) will be reviewed. This will then allow a discussion regarding how best to intervene to ensure that the best interests of traumatized individuals who are passing through complex criminal justice systems are met.

## Afternoon - Half Day Workshops

### STAIR (Skill Training in Affective and Interpersonal Regulation) narrative therapy for adolescents

#### M. Cloitre^1,2^

^1^Research National Center for PTSD – Dissemination and Training Division, Palo Alto, CA, USA; ^2^Psychiatry and Child & Adolescent Psychiatry, NYU Langone Medical Center, NY, USA

Childhood physical or sexual abuse, neglect and other forms of maltreatment can result in developmental injuries that result in emotion regulation difficulties, a negative self-concept, and problems in relational and social functioning. STAIR (Skill Training in Affective and Interpersonal Regulation) Narrative Therapy (SNT) is a phase-based treatment guided by the principle that recovery requires not only the emotional processing of the traumatic experiences but also the rehabilitation of a positive self-concept as well as emotional, social and interpersonal capacities needed for effective living. This workshop will review SNT interventions as applicable to adolescents with example case presentations and experiential exercises. The initial treatment module, Skill Training in Affective and Interpersonal Regulation (STAIR), focuses on building and strengthening emotional awareness and regulation and more diverse, flexible and compassionate “working models” of self in relationship to others. The second module introduces the narration of trauma memories and their organization into a compassionate autobiography. SNT has been demonstrated to provide significant and clinically substantial relief from PTSD as well as improvement in emotion management and interpersonal functioning in adolescents.

### Challenge and facilitation of trauma exposure therapy in survivors with dissociative reactions

#### M. Schauer^1^ and Martina Ruf-Leuschner^2^

^1^Center of Excellence for Psychotraumatology, University of Konstanz, Konstanz, Germany; ^2^University of Konstanz, Konstanz, Germany

Dissociation is a serious obstacle in the processing of traumatic experiences in survivors suffering from the consequences of multiple and complex stressors. An aetiological model of dissociation, derived from the repertoire of psychophysiological defense in response to threatening experiences, provides pragmatic intervention techniques to facilitate trauma-focused treatment. Extremely dangerous conditions evoke an evolutionary preserved defense cascade of survival responses, which escalate in relation to proximity and perceived characteristics of threat and perpetrator. First, there is the sympathetically dominated alarm response (flight and fight), which culminates in tonic immobility (fright), characterized by high dual autonomic tone. If there is no escape possibility, parasympathetic arousal remains high, while loss of sympathetic dominance will lead to dissociative loss of function and eventually fainting (flag and faint). We suggest that trauma treatment must therefore be aware of patient's reactions on two dimensions: those with peritraumatic sympathetic activation versus those, who went down the whole defense cascade, which leads to parasympathetic dominance during the trauma and a corresponding replay of dissociative responding and even fainting, when reminded. The differential management of dissociative stages and other important treatment implications will be presented.

